# Comparison of Scaffolds Fabricated via 3D Printing and Salt Leaching: In Vivo Imaging, Biodegradation, and Inflammation

**DOI:** 10.3390/polym12102210

**Published:** 2020-09-26

**Authors:** Doo Yeon Kwon, Joon Yeong Park, Bun Yeoul Lee, Moon Suk Kim

**Affiliations:** Department of Molecular Science and Technology, Ajou University, Suwon 16499, Korea; kdy@ajou.ac.kr (D.Y.K.); pjy16@ajou.ac.kr (J.Y.P.); bunyeoul@ajou.ac.kr (B.Y.L.)

**Keywords:** scaffold, salt-leaching, printing, in vivo imaging, biodegradation

## Abstract

In this work, we prepared fluorescently labeled poly(ε-caprolactone-ran-lactic acid) (PCLA-F) as a biomaterial to fabricate three-dimensional (3D) scaffolds via salt leaching and 3D printing. The salt-leached PCLA-F scaffold was fabricated using NaCl and methylene chloride, and it had an irregular, interconnected 3D structure. The printed PCLA-F scaffold was fabricated using a fused deposition modeling printer, and it had a layered, orthogonally oriented 3D structure. The printed scaffold fabrication method was clearly more efficient than the salt leaching method in terms of productivity and repeatability. In the in vivo fluorescence imaging of mice and gel permeation chromatography of scaffolds removed from rats, the salt-leached PCLA scaffolds showed slightly faster degradation than the printed PCLA scaffolds. In the inflammation reaction, the printed PCLA scaffolds induced a slightly stronger inflammation reaction due to the slower biodegradation. Collectively, we can conclude that in vivo biodegradability and inflammation of scaffolds were affected by the scaffold fabrication method.

## 1. Introduction

Regenerative medicine has recently attracted significant interest for the repair of damaged human tissues and organs [1]. An appropriate combination of a three-dimensional (3D) scaffold, cells, such as stem cells, and biologically active molecules is needed to regenerate damaged human tissues and organs [2]. It is particularly important to fabricate 3D scaffolds that perfectly restore a damaged organ or tissue.

Salt leaching is among the most common methods used to fabricate 3D scaffolds [3,4,5]. Biocompatible materials that are currently used to fabricate 3D scaffolds via the salt-leaching method include natural biomaterials, such as collagen, hyaluronic acid, and gelatin, as well as synthetic materials, such as polyester and polyethylene glycol (PEG) [6,7,8]. Synthetic polyesters can be precisely designed to have specific molecular weights and compositions [9,10,11,12]. In addition, synthetic polyesters can be produced in large quantities with little or no batch-to-batch variation. Synthetic polyesters, including poly(ε-caprolactone) (PCL), polylactic acid (PLA), polyglycolic acid (PGA), and various copolymers, are frequently used as biomaterials to fabricate scaffolds via the salt-leaching method [13,14,15]. Although salt leaching is generally a facile fabrication method, it is difficult to precisely control the 3D structure of the scaffolds. It is also necessary to completely remove the solvents and salts after fabrication [16].

**Figure 1:**
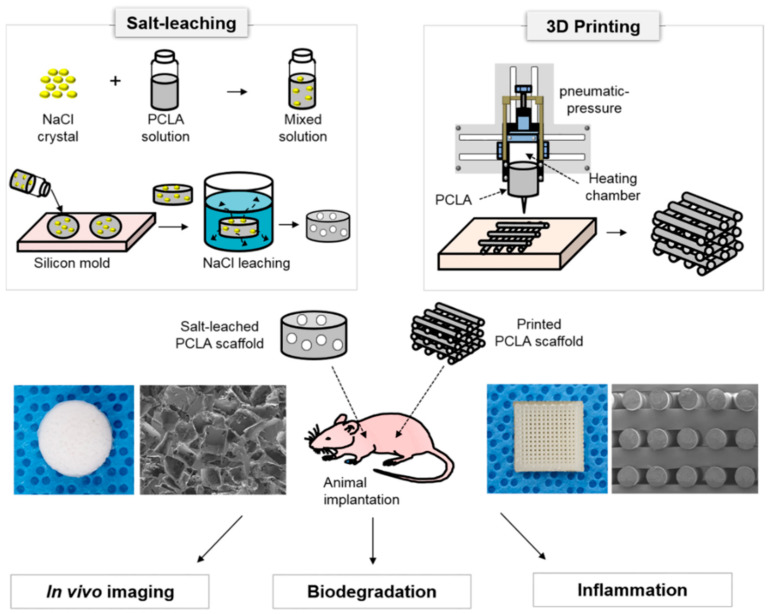
Schematic illustration for the comparison of scaffolds fabricated via 3D printing and salt leaching: In vivo imaging, biodegradation, and inflammation (the images were drawn by J.Y.P. and M.S.K. in the Adobe Photoshop 7.0 software).

3D printing has been proposed as a way to precisely regenerate human tissues and organs [17,18]. Various printers have been developed to fabricate 3D scaffolds. Thermal melting printers are referred to as fused deposition modeling (FDM) printers, and they have come into widespread use [19]. FDM printers can produce 3D scaffolds without organic solvents by transforming flowing bioinks into solid structures at an ambient temperature. Polyesters are particularly useful as FDM printing bioinks for the fabrication of 3D scaffolds [20,21,22]. We recently prepared biodegradable polyesters with well-defined compositions that contained PLA, PGA, and PCL segments. We also used polyester bioinks and an FDM printer to prepare 3D scaffolds. We compared the in vivo biodegradation and inflammation-inducing properties of the scaffolds at the implantation sites (Figure 1) [23,24,25].

To our knowledge, few studies have compared in vivo biodegradability and inflammation of scaffolds using the scaffold fabrication method. Therefore, we prepared poly(ε-caprolactone-ran-lactic acid) (PCLA) as a biomaterial and used salt leaching and 3D printing methods to fabricate 3D scaffolds. We fluorescently labeled PCLA to perform in vivo imaging of the salt-leached and printed scaffolds in real time. We compared the efficiencies of salt leaching and 3D printing for the fabrication of PCLA scaffolds. Additionally we evaluated in vivo biodegradation and inflammation to compare the in vivo utilization of the salt-leached and printed PCLA scaffolds.

## 2. Materials and Methods

### 2.1. Characterization

The structures of PCLA copolymer and degraded PCLA were measured by ^1^H nuclear magnetic resonance (NMR) spectra of a Mercury Plus 400 NMR system (Varian, Palo Alto, CA, USA) using CDCl_3_ with tetramethylsilane as an internal standard. The melting temperature (*T_m_*) of PCLA copolymer was determined by DSC (200 F3, NETSZCH, Bonn, Germany). The sample was cooled from 25 to −80 °C at a rate of 10 °C/min, and then, the sample was heated from −80 to 200 °C for the final analysis.

### 2.2. Synthesis of the PCLA Copolymer

Monomethoxypolyethylene glycol [MPEG, a number average molecular weight (*M_n_*) is 750 g/mol, Aldrich; St. Louis, MO, USA] and stannous octoate (Aldrich; St. Louis, MO, USA) were used as received. ε-Caprolactone (CL) was distilled in the presence of CaH_2_ by using reduced pressure. l-lactide (LA) (Boehringer Ingelheim; Ingelheim, Germany) was recrystallized twice using ethyl acetate. All reactions were performed in glassware dried before use by heating it in a vacuum oven and flushed with a dry nitrogen stream. MPEG (0.012 g, 0.016 mmol) and toluene (80 mL) were added to a round flask. Azeotropic distillation was performed to remove the water from the MPEG solution. Toluene was then removed via distillation to obtain a final volume of 30 mL. Then, LA (4.46 g, 31.0 mmol) and CL (3.54 g, 31.0 mmol) were added to the MPEG solution at room temperature under a nitrogen atmosphere. A 0.1 M stannous octoate solution in dried toluene (0.19 mL) was added to the solution, and the mixture was stirred at 130 °C for 24 h. The reaction mixture was poured into a 4/1 (*v/v*) mixture of *n*-hexane and ethyl ether to precipitate the polymer. The supernatant was decanted, and the polymer was dissolved in CH_2_Cl_2_. The solvent of the resulting mixture was filtered and removed by a rotary evaporator and then dried in vacuo to provide a colorless polymer. The ratios and molecular weights of PLA and PCL segments were determined from the ^1^H NMR spectrum by comparing the intensities of methylene proton signals of the MPEG standard (750 g/mol), PLA, and PCL at δ 5.18, 4.15, and 2.36, respectively.

### 2.3. Labeling PCLA with Fluorescein Isothiocyanate (PCLA–F)

PCLA (4 g, 0.008 mmol) and toluene (80 mL) were added to a flask. Water in the PCLA solution was removed via azeotropic distillation. Then, toluene was removed via distillation to obtain a final volume of 30 mL. Fluorescein isothiocyanate (FITC, 4.2 mg, 0.01 mmol) was added to the PCLA solution at room temperature under a nitrogen atmosphere. A 0.1 M stannous octoate solution in dried toluene (0.1 mL) was added, and the reaction mixture was stirred at 130 °C for 24 h. The mixture was then poured into a 2/1/1 (*v/v/v*) mixture of *n*-hexane, ethyl ether, and methanol to precipitate the polymer. The supernatant was decantated, and the polymer was dissolved in CH_2_Cl_2_. The resulting solution was filtered and concentrated on a rotary evaporator and then dried in vacuo to give PCLA-F in 85% yield.

### 2.4. Labeling PCLA with Rhodamine Isothiocyanate (PCLA–R)

PCLA-R was prepared using rhodamine isothiocyanate (RITC, 5.7 mg, 0.01 mmol) as described for PCLA-F and obtained in 87% yield.

### 2.5. Salt-Leaching Fabrication of a PCLA Scaffold

NaCl was sieved to isolate particles with diameters of 200–250 μm, and the obtained salt was used as a porogen. PCLA-F or PCLA-R was individually dissolved in methylene chloride (CH_2_Cl_2_), and then, the sieved NaCl particles (90 wt % NaCl) were added to each PCLA-F or PCLA-R solution. The gel-like PCLA-F/NaCl or PCLA-R/NaCl slurry was gently mixed using a spatula and poured into a circular silicone mold (10 diameter × 5 mm length). The circular silicone mold loaded with the PCLA-F/NaCl or PCLA-R/NaCl slurry was pressed using an AJP-12 hydraulic press (Anjeon Hydraulic Machinery; Korea) for 3 h under 700 kg/cm^2^ of pressure. The molded slurry was dried at room temperature for 24 h. The PCLA scaffold was detached from the mold and washed in deionized water for 24 h at 100 rpm to remove the excess NaCl. After NaCl removal, the scaffold was lyophilized for 48 h at 80 °C. The obtained PCLA scaffold was stored in a vacuum desiccator until use.

### 2.6. Fabrication of a PCLA Scaffold via 3D Printing

A SFF 3D plotter (ProtekKorea; Daejeon, Korea) was used for 3D printing. It comprised a plotting system equipped with a heating jacket and a stainless steel cylinder with a micronozzle. The micronozzle had an internal diameter of 300 µm, and it was moved by an air dispenser in the direction of the *xyz* stage. The plotting system was controlled using the Scaffold Path Generation SW computer software (Korea Institute of Machinery and Materials; Daejeon, Korea). PCLA was added to the stainless steel barrel of the heating jacket and melted at 180 °C. The melted PCLA was extruded from the micronozzle with pressurized air (100 kPa). The computer-designed PCLA scaffold was 10 mm in width, 10 mm in depth, and 5 mm in height. The orthogonally oriented scaffold was fabricated layer by layer with 100-µm pores in its vertical cross section. The first PCLA layer was deposited in a series of parallel lines along the *y-*direction, and the second layer was deposited parallel to the *x-*direction. The third and fourth layers were applied using the same deposition procedure. The fabrication process afforded a solid PCLA scaffold at an ambient temperature.

### 2.7. Animal Implantation Surgery

The protocols used in this study were approved by the Ajou University School of Medicine Institutional Animal Experiment Committee (approval no. 2012-0004). Experiments were performed using eight-week-old Sprague-Dawley rats weighing 320–350 g and six-week-old male nude mice weighing 20–22 g in accordance with the approved guidelines. The rats were divided into two groups. Salt-leached scaffolds were implanted in one group (*n* = 9), and printed PCLA scaffolds were implanted in the other (*n* = 9). All scaffolds were implanted subcutaneously on the dorsal sides of the rats and mice after they were anesthetized with Zoletil^®^ and Rompun^®^ (1:1) at a dose of 1.5 mL/kg. The salt-leached and printed PCLA scaffolds were allowed to develop. The rats were euthanized individually four, eight, and sixteen weeks after implantation (three rats per each time point), and the salt-leached and printed PCLA scaffolds were excised from the implantation sites. Three scaffolds removed from rats at each time point were used for each GPC, SEM, and histological analysis, respectively.

### 2.8. In Vivo Fluorescence Imaging

Four, eight, and sixteen weeks after implanting the salt-leached and printed PCLA scaffolds in mice (three mice per group), in vivo fluorescence was measured at 515 nm (λ_em_) with excitation at 470 nm using a FO ILLUM PL-800 fluorescence imaging system (Edmund Optics; Barrington, NJ, USA) equipped with a 150 W EKE quartz halogen light and an OG515 glass filter (Newport Corporation in Irvine, CA, USA). A charge-coupled device was used for digitization, and the fluorescence images were processed using the AxioVision Rel. 4.8 software package. The fluorescence signal was established to minimize the auto-fluorescence of mice on day 0. Each fluorescence signal intensity of mice measured at each time point was compared to the fluorescence signal intensity measured on day 0. At each time point, the fluorescence was determined using the ImageJ software, version 1.44 (National Institutes of Health; Bethesda, MD, USA). All fluorescence images were normalized identically for all experimental conditions. The degradation ratio from the fluorescence signal was defined as follows: Degradation (%) = [(fluorescence signal intensity on day 0-fluorescence signal intensity at each time point) ÷ (fluorescence signal intensity on day 0)] × 100.

### 2.9. Scanning Electron Microscopy (SEM)

The salt-leached and printed PCLA scaffolds were removed from rats four, eight, and sixteen weeks after implantation. The removed scaffolds were quickly immersed in liquid nitrogen to minimize structural changes and then freeze-dried at −75 °C. To measure SEM, the dried scaffolds were coated with a thin layer of gold using a 108 Auto plasma sputter apparatus (Cressington; Redding, CA, USA) under argon gas and then observed using a JSM-6380 SEM (JEOL; Tokyo, Japan).

### 2.10. Characterization of In Vivo Biodegradation

For in vivo biodegradation measurements, the excised, salt-leached, and printed PCLA scaffolds were first placed in test tubes. CH_2_Cl_2_ (1 mL) was added to each tube to dissolve PCLA in the implant, and 1 mL of distilled water (DW) was added to disperse the tissue. The resulting mixtures were sonicated for 90 min at 25 °C, and the tubes were centrifuged at 10,000 rpm for 5 min. Then, CH_2_Cl_2_ layers were collected and CH_2_Cl_2_ was removed. The remaining portions containing the degraded PCLA components were freeze-dried until the residues reached constant weights. The molecular weights of the degradation products were determined via gel permeation chromatography (GPC). The PCLA components were quantified by analyzing the characteristic peaks in the NMR spectra.

### 2.11. Histological Analysis

Rats were euthanized four, eight, and sixteen weeks following implantation. The implants were removed from the subcutaneous dorsa and dissected individually. The implants were immediately fixed with 10% formalin and decalcified for 8 h using decalcifying solution lite (Sigma; St. Louis, MO, USA). The fixed tissues were then dehydrated and embedded in paraffin. The embedded specimens were sectioned into 4-μm slices, which were stained with hematoxylin and eosin (H&E). ED1 monoclonal antibody was used to detect macrophages. For H&E staining, the hydrated slides were washed under running tap water and then stained with hematoxylin. After 3 min, the slides were washed with DW and incubated in Scott’s water. After 3 min, the stained slides were washed with DW and then dyed with eosin for 2 min and washed with DW. The slides were incubated in 70% ethyl alcohol. After 5 min, the slides were washed with DW and then dried at room temperature. Finally, the dried slides were fixed and mounted with a mounting medium (Pure Chemicals; Tokyo, Japan).

Hydrated slides were also incubated for 10 min at 120–130 °C in a citrate buffer (Sigma; St. Louis, MO, USA) with 4,6-diamino-2-phenylindole dihydrochloride (DAPI), which was used as a marker for macrophages (ED1). The slides were washed in tris-buffered saline (TBS) for 5 min and then heated for 30 min at 37 °C in PBS containing 5% bovine serum albumin (BSA; Roche, Penzberg, Germany). The slides were incubated at 4 °C for 16 h with an anti-BrdU antibody (DAKO; Glostrup, Denmark) in a 1% BSA solution (650:1). The slides were washed three times with TBS and incubated with 1:200 goat anti-rabbit Alexa Fluor^®^ 594 secondary antibody (Invitrogen; Gaithersburg, MD, USA) for 3 h in the dark at room temperature. The slides were washed again with PBS. After 5 min, the slides were counterstained with DAPI (Sigma; St. Louis, MO, USA) in DW (1:1000) for 10 min and mounted with Pro-Long Gold Antifade Reagent and DAPI (Life technologies; Grand Island, NY, USA).

Axio Imager A1 (Carl Zeiss Microimaging GmbH; Göttingen, Germany) was used to obtain immunofluorescent images. The obtained images were analyzed using the AxioVision software Rel. 4.8 (Carl Zeiss Microimaging GmbH; Göttingen, Germany). ED1-positive areas in each specimen were measured at three randomly selected positions using the ImageJ software package (National Institutes of Health; Bethesda, MD, USA). All data were reported as the mean with standard deviation (SD).

### 2.12. Statistical Analysis

All data were reported as the mean with standard deviation (SD). A one-way analysis of variance (ANOVA) with Bonferroni correction was performed for the results using the SPSS 12.0 software package (SPSS Inc.; Chicago, IL, USA).

## 3. Results and Discussion

### 3.1. Synthesis of the PCLA Copolymer

In our previous work, we prepared PCLA with various molecular weights and compositions [23,24,25]. The degradation of PCLA can occur by hydrolytic scission of ester bond linkages over a period ranging from days to a few weeks. From the PCLA series, we chose PCLA with a 1:1 molar ratio of CL and LA and a molecular weight of 500 kD, showing the half-life of molecular weight of eight weeks (because we designed bone regeneration using printed PCLA scaffolds as a part of other future work).

**Figure 2:**
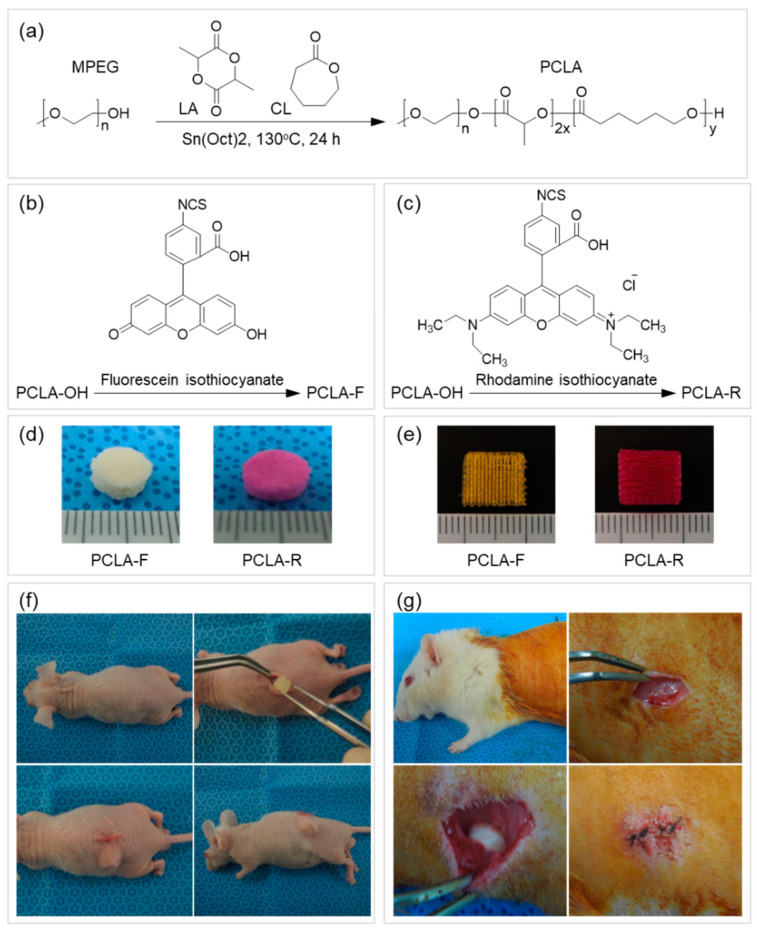
Schematic illustration of (**a**) poly(ε-caprolactone-ran-lactic acid) (PCLA), (**b**) PCLA-fluorescein isothiocyanate (PCLA-F), and (**c**) PCLA-rhodamine isothiocyanate (PCLA-R) preparation and images of (**d**) the salt-leached and (**e**) the printed PCLA-F and PCLA-R scaffolds. Subcutaneous implantation of salt-leached and printed PCLA-F and PCLA-R scaffolds on the dorsal sides of (**f**) nude mice for fluorescence imaging and (**g**) Sprague-Dawley (SD) rats to assess biodegradation and inflammation.

Herein, PCLA was synthesized via ring-opening polymerization of CL and LA using MPEG (750 g/mol) as an initiator in the presence of stannous octoate at 130 °C for 24 h (Figure 2a). The polymerization product was isolated after precipitation, and a colorless PCLA copolymer was obtained in a nearly quantitative yield. The PCLA copolymer contained CL and LA in a 1:1 ratio, and a molecular weight of ~500 kD was confirmed via ^1^H NMR spectroscopy using MPEG as a standard. The PCLA copolymer generated monomodal gel permeation chromatograms. To perform in vivo fluorescence imaging in real time, the PCLA copolymer was labeled with FITC and RITC to generate green and red fluorescence, respectively (Figure 2b,c). PCLA-F and PCLA-R were isolated in the above 85% yields.

### 3.2. Fabrication of the Salt-Leached and Printed PCLA Scaffolds

Several previous studies have examined the pore formation of the salt-leached scaffold according to the concentration of salts [26]. In this work, we used NaCl with 200–250 μm as porogen, but we could not adjust the exact pore structure in the processing of salt-leached scaffold fabrication. The PCLA copolymer was placed in a circular disk-type silicone mold to fabricate the salt-leached PCLA scaffold, which had an irregular interconnected 3D structure. Preparation of the PCLA scaffold using the salt-leaching method was successful and facile. However, the porous, interconnected structures of the salt-leached PCLA scaffolds can be affected by the particle size of the salt and subsequent leaching from the scaffolds [26,27]. Therefore, the salt-leaching procedure can affect the uniformity of the scaffolds and the reproducibility of fabrication because the pore structure and porosity depended on the concentration of porogen in the previous work [26,27].

To use the PCLA copolymer for FDM printing, the PCLA copolymer was first melted and examined by performing differential scanning calorimetry (DSC). Phase transitions at 140 and 53 °C were attributable to PLA and PCL segments, respectively. This confirmed that the PCLA copolymer would melt readily at 180 °C in the heating jacket of the FDM printer. The PCLA copolymer flowed evenly from the micronozzle of the heating jacket. The PCLA scaffold was printed in layers with orthogonal orientations on a stainless plate. The printed PCLA scaffold was 10 mm wide, 10 mm deep, and 5 mm high. It had a strand size of 200 µm and contained 100-µm pores in its vertical cross section.

The printed PCLA scaffold was fabricated in approximately 3 min. Salt removal and drying of the salt-leached scaffold took more than 3 days. After comparing the salt-leaching and printing methods, printing was clearly more efficient than the salt-leaching method for the fabrication of PCLA scaffolds in terms of productivity and repeatability.

### 3.3. In Vivo Fluorescence Imaging of Salt-Leached and Printed PCLA Scaffolds

**Figure 3:**
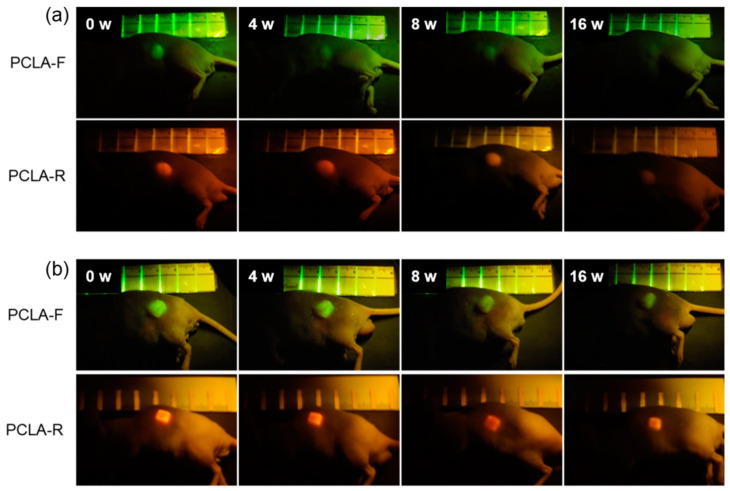
In vivo fluorescence images of nude mice recorded over 16 weeks following the implantation of (**a**) salt-leached and (**b**) printed PCLA-F and PCLA-R scaffolds.

To assess biodegradation, the salt-leached and printed PCLA-F and PCLA-R scaffolds were implanted subcutaneously for in vivo fluorescence imaging (Figure 2c). The implantation procedure and implanted scaffolds were tolerated well by all nude mice. Green and red fluorescence images of the nude mice were acquired after subcutaneous implantation of the salt-leached and printed PCLA-F and PCLA-F scaffolds (Figure 3). Intense green and red fluorescence at the implantation sites of all salt-leached and printed PCLA-F and PCLA-F scaffolds was immediately observed. The intensity of green and red fluorescence in the images gradually decreased over the following 16 weeks. Only green fluorescence imaging was performed in the next experiment.

**Figure 4:**
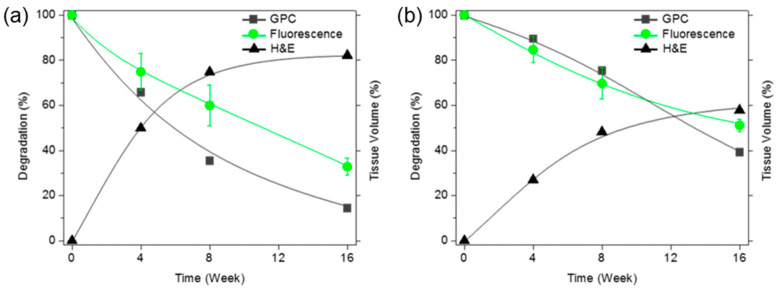
Time since implantation versus the molecular weights of the degraded scaffolds based on the maximum gel permeation chromatography (GPC) signals (-■-) and green fluorescence intensities (-●-) of (**a**) salt-leached and (**b**) printed PCLA-F scaffolds measured over 16 weeks. The tissue volumes were calculated after H&E staining (-▲-).

The intensity of green fluorescence following implantation was calculated from the fluorescence images (Figure 3) and plotted over time. This is represented by the green curves in Figure 4a,b. The fluorescence intensity gradually decreased. The salt-leached PCLA scaffold excised on week 4 had degraded by 25%, and 68% of the initial fluorescence intensity had disappeared by week 16 (Figure 4a). This indicated that the salt-leached PCLA scaffolds degraded gradually over 16 weeks. The printed PCLA scaffolds showed 15% degradation by week 4, 31% by week 8, and 49% by week 16 (Figure 4b). The printed PCLA scaffolds thus degraded more slowly than the salt-leached PCLA scaffolds.

Based on the measured fluorescence intensity, the PCLA scaffolds gradually degraded in vivo during the experimental period. The fluorescence imaging results confirmed that degradation of the PCLA scaffolds could be monitored noninvasively. In addition, we found that the salt-leached and printed PCLA scaffolds biodegraded at different rates.

### 3.4. Morphologies of the Salt-Leached and Printed PCLA Scaffolds

**Figure 5:**
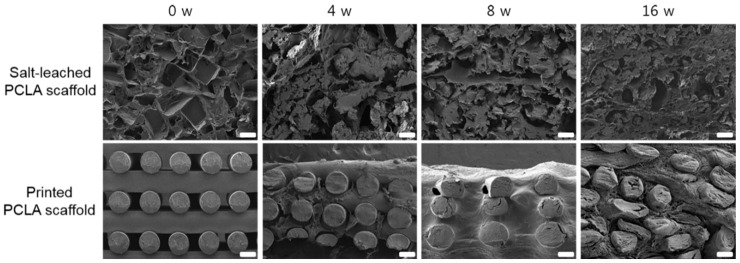
Cross-sectional SEM images of salt-leached and printed PCLA scaffolds removed from SD rats four, eight, and sixteen weeks after implantation. Scale bars = 100 μm.

The salt-leached and printed PCLA scaffolds removed from the rats four, eight, and sixteen weeks after implantation were examined via SEM (Figure 5). The SEM images of the salt-leached PCLA scaffolds showed an irregular pore 3D structure. As the implantation time increased, the salt-leached PCLA scaffolds revealed disintegration of their inner structure. The degradation of PCLA copolymer was generally considered as the process of bulk erosion even though there are many variables that influence the degradation process. Therefore, it was explained that the salt-leached PCLA scaffolds with irregular pore structures underwent a rapid bulk erosion process and then induced fast collapse of inner structure of the scaffold, implying fast degradation.

Meanwhile, the printed PCLA scaffold excised on week 4 maintained its regular strands. It retained its cylindrical form but appeared slightly flattened. Strand fragments in the printed PCLA scaffold were interspersed with the connective tissue. By week 16, strands in the printed PCLA scaffold had gradually collapsed, implying slow degradation. This indicated that the printed PCLA scaffolds remained morphologically intact for a longer period of time due to slower in vivo biodegradation.

### 3.5. In Vivo Degradation of Salt-Leached and Printed PCLA Scaffolds

**Figure 6:**
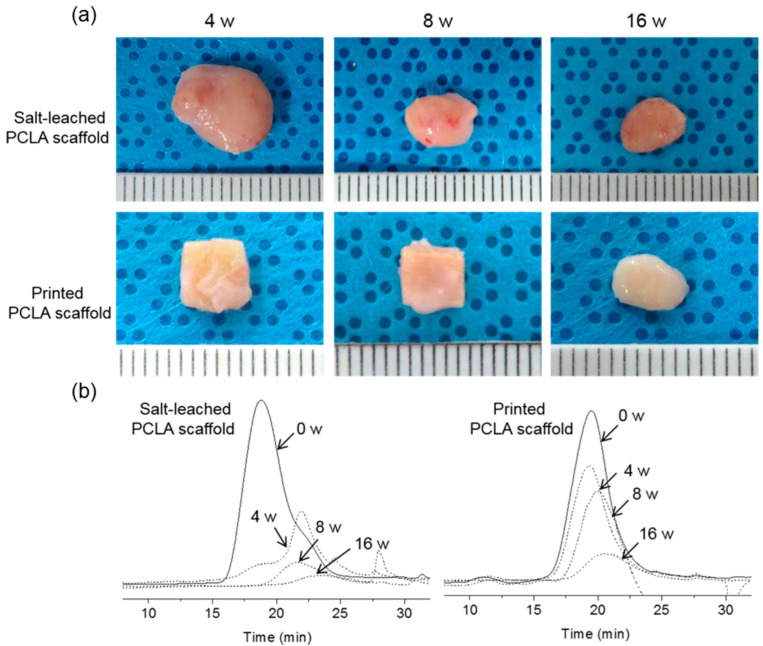
(**a**) Photographs and (**b**) changes in the GPC spectra of salt-leached and printed PCLA scaffolds removed from SD rats four, eight, and sixteen weeks after implantation.

In vivo degradation of the salt-leached and printed PCLA scaffolds in the rats was assessed four, eight, and sixteen weeks following implantation (Figure 2g). Both the implantation procedure and the implanted scaffolds were tolerated well by all rats. The salt-leached and printed PCLA scaffolds were allowed to develop for 16 weeks in vivo and were then excised. All salt-leached and printed PCLA scaffolds were easily identified and isolated from the tissues surrounding the implantation sites. Salt-leached and printed PCLA scaffolds were excised four, eight, and twelve weeks after implantation and photographed (Figure 6a). Thin, fibrous capsules containing fibroblasts and vascular vessels developed around the salt-leached and printed PCLA scaffolds, which was consistent with the organization process of new tissue formation. However, fibrous capsules and vascular vessels were observed in a smaller amount on printed PCLA scaffolds than on the salt-leached PCLA scaffolds. This suggests that the regular and less biodegradable strands of the printed PCLA scaffolds make it relatively difficult to grow vascular vessels on surface of and inside these scaffolds.

Therefore, the extent of degradation was monitored via GPC. After implantation, peaks in the GPC chromatograms of all the salt-leached and printed PCLA scaffolds shifted over time to lower molecular weights corresponding to degradation products (Figure 6b). The molecular weights of the scaffolds following in vivo degradation were determined from the most intense GPC peaks. Degradation (%) was calculated from the relative ratio of the molecular weights measured at the time of implantation and the day of excision. The intensity of GPC peaks decreased gradually, indicating gradual degradation (Figure 4a,b).

The salt-leached PCLA scaffolds showed several peaks of oligomers or degraded molecular weights assignable degraded peaks. The degraded peaks increased over time and showed more extensive degradation at 12 weeks. Meanwhile, the printed PCLA scaffolds showed little changes in the GPC peak, indicating little degradation. The salt-leached PCLA scaffolds excised on week 4, 8, and 16 retained 34%, 65%, and 85% of their original molecular weights, respectively. Biodegradation of the printed PCLA scaffolds was more gradual. Biodegradation was 11% on week 4, 25% on week 8, and 61% on week 16. Evidently, the printed PCLA scaffolds degraded more slowly than the salt-leached PCLA scaffolds as the implantation time increased.

The results obtained by SEM, GPC, and fluorescence imaging were in good agreement in terms of showing that the salt-leached PCLA scaffolds extensively degraded but the printed PCLA scaffolds showed markedly less and slower degradation at all-time points. Collectively, the observed differences in biodegradation were attributed to differences between the structures of the salt-leached and printed PCLA scaffolds.

**Figure 7:**
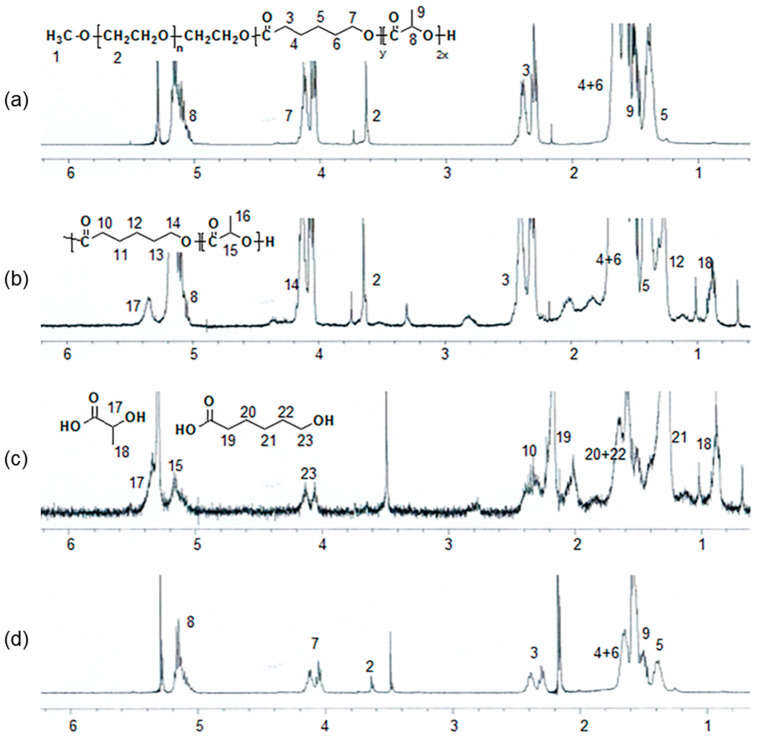
^1^H NMR spectra of a PCLA scaffold (**a**) before in vivo degradation and (**b**–**d**) eight weeks after implantation; (**b**) the crude mixture; (**c**) components isolated in n-hexane and ethyl ether; and (**d**) insoluble components.

Changes in the ^1^H NMR spectra of the printed PCLA scaffolds were observed after eight weeks (Figure 7). The spectra also contained peaks that were characteristic of the degraded PCLA copolymer and MPEG after separation in n-hexane and ether. Signals *17* and *18* could be assigned to lactic acid. Signals *23* and *2* were assigned to 6-hydroxyl hexanoic acid and MPEG, respectively.

### 3.6. Inflammation at the Salt-Leached and Printed PCLA Scaffold Implantation Sites

To assess the biocompatibility of the salt-leached and printed PCLA scaffolds, the implanted scaffolds were excised at various post-implantation times, sectioned, and examined after histological and immunohistochemical staining.

**Figure 8:**
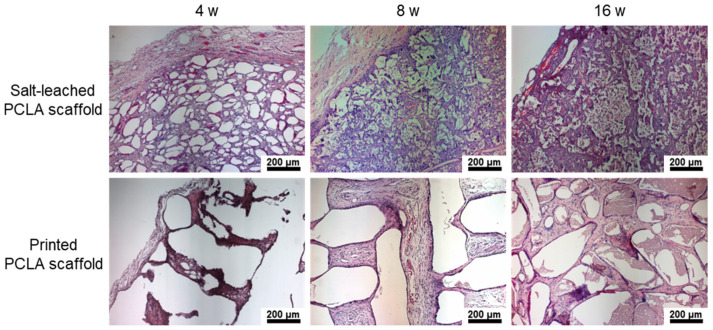
H&E staining images of salt-leached and printed PCLA scaffolds excised four, eight, and sixteen weeks after implantation. Blood vessels are indicated by arrows. Scale bars = 200 μm.

In the H&E staining of salt-leached scaffolds, the pores were observed at 4 weeks, and then, a crushed and degraded pore structure was noted to develop gradually. Conversely, the printed PCLA scaffolds showed empty space due to the non-biodegraded cylindrical forms of strands of printed PLCA. Additionally, H&E staining revealed the penetration of host cells and dense accumulations of tissue around each salt-leached and printed PCLA scaffold (Figure 8), and new blood vessels were visible in the scaffold.

**Figure 9:**
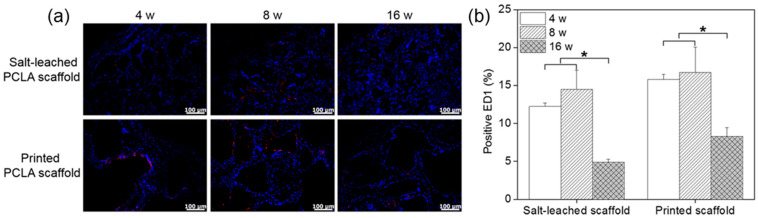
(**a**) Immunofluorescence ED1 staining images and (**b**) number of ED1-positive cells on salt-leached and printed PCLA scaffolds four, eight, and sixteen weeks after implantation. Scale bars = 100 μm, * *p* < 0.001.

The implantation of scaffold can change the host response as a foreign body reaction [28,29]. It has been proposed that mechanical properties of the interface between a scaffold and its surrounding tissues are critical for determining the host response; however, the underlying inflammatory mechanism is still poorly understood. Among several inflammatory cells such as neutrophils, macrophages, and monocytes, macrophages act primarily at the interface between the host tissue and implanted scaffold. Therefore, to assess the local biocompatibility of the salt-leached and printed PCLA scaffolds in and around the implantation sites, tissues were stained with the macrophage markers ED1 and DAPI to evaluate the extent of host cell infiltration and inflammatory cell accumulation (Figure 9a). DAPI staining (blue) revealed the presence of many host cells around the salt-leached and printed PCLA scaffolds. ED1 staining (red) confirmed the accumulation of macrophages on the scaffold surfaces and in the surrounding tissues (Supplementary Information Figure S1). Macrophages infiltrated the implantation sites to remove the salt-leached and printed PCLA scaffolds.

ED1-positive cells in the tissues were counted and normalized relative to the total stained areas to determine the extent of inflammation (Figure 9b). Four weeks after implantation, 12–15% of cells in the tissues near all of the salt-leached and printed PCLA scaffolds were ED1-positive. This percentage gradually increased to 14–16% in rats within eight weeks of subcutaneous implantation and then decreased to 5–8% by week 16. The printed PCLA scaffolds contained slightly more ED1-positive cells than the salt-leached PCLA scaffolds. We surmised that the printed PCLA scaffolds as foreign bodies induced a slightly stronger inflammation reaction because they remained in the body longer [28,29].

## 4. Conclusions

We successfully fabricated PCLA scaffolds using salt-leaching and printing methods. Compared with the salt-leaching method, printing was the more efficient scaffold fabrication method in terms of productivity and repeatability. The in vivo imaging and GPC analysis to assess in vivo biodegradation showed more extensive degradation in salt-leached scaffolds than in printed scaffolds because of the irregular structure of the salt-leached PCLA scaffolds, which induced a rapid bulk erosion process. In the inflammation reaction, the printed PCLA scaffolds induced a slightly stronger inflammation reaction due to the slow biodegradation. Our results suggest that biodegradation and inflammation were affected by the scaffold fabrication method. Therefore, a further investigation for bone regeneration using the salt-leached and printed PCLA scaffolds will be performed in future studies.

## Supplementary Materials

The following are available online at https://www.mdpi.com/2073-4360/12/10/2210/s1, Figure S1: Immunofluorescence ED1 staining images on salt-leached and printed PCLA scaffolds four, eight, and sixteen weeks after implantation. Scale bars = 100 μm.

## References

[1] Dzobo K., Thomford N.E., Senthebane D.A., Shipanga H., Rowe A., Dandara C., Pillay M., Motaung K.S.C.M. (2018). Advances in regenerative medicine and tissue engineering: Innovation and transformation of medicine. Stem Cells Int..

[2] Zhang L., Yang G., Johnson B.N., Jia X. (2019). Three-dimensional (3D) printed scaffold and material selection for bone repair. Acta Biomater..

[3] Przekora A. (2019). The summary of the most important cell-biomaterial interactions that need to be considered during in vitro biocompatibility testing of bone scaffolds for tissue engineering applications. Mater. Sci. Eng. C Mater. Biol. Appl..

[4] Du Y., Guo J.L., Wang J., Mikos A.G., Zhang S. (2019). Hierarchically designed bone scaffolds: From internal cues to external stimuli. Biomaterials.

[5] Sofi H.S., Ashraf R., Beigh M.A., Sheikh F.A. (2018). Scaffolds fabricated from natural polymers/composites by electrospinning for bone tissue regeneration. Adv. Exp. Med. Biol..

[6] Cai Z., Wan Y., Becker M.L., Long Y.Z., Dean D. (2019). Poly(propylene fumarate)-based materials: Synthesis, functionalization, properties, device fabrication and biomedical applications. Biomaterials.

[7] Fuchs S., Shariati K., Ma M. (2019). Specialty tough hydrogels and their biomedical applications. Adv. Health Mater..

[8] Park K.M., Park K.D. (2018). In situ cross-linkable hydrogels as a dynamic matrix for tissue regenerative medicine. Tissue Eng. Regen. Med..

[9] Kim D.Y., Kwon D.Y., Kwon J.S., Kim J.H., Min B.H., Kim M. (2015). Stimuli-responsive injectablein situ-forming hydrogels for regenerative medicines. Polym. Rev..

[10] Tedesco M.T., Di Lisa D., Massobrio P., Colistra N., Pesce M., Catelani T., Dellacasa E., Raiteri R., Martinoia S., Pastorino L. (2018). Soft chitosan microbeads scaffold for 3D functional neuronal networks. Biomaterials.

[11] Liu X., Fan X., Jiang L., Loh X.J., Wu Y.L., Li Z. (2018). Biodegradable polyester unimolecular systems as emerging materials for therapeutic applications. J. Mater. Chem. B.

[12] Nazir N.M., Zulkifly A.H., Khalid K.A., Zainol I., Zamli Z., Sha’Ban M. (2019). Matrix production in chondrocytes transfected with sex determining region Y-Box 9 and telomerase reverse transcriptase genes: An in vitro evaluation from monolayer culture to three-dimensional culture. Tissue Eng. Regen. Med..

[13] Hauptmann N., Lian Q., Ludolph J., Rothe H., Hildebrand G., Liefeith K. (2019). Biomimetic designer scaffolds made of D,L-lactide-ɛ-caprolactone polymers by 2-photon polymerization. Tissue Eng. Part. B Rev..

[14] Qian Y., Zhou X., Zhang F., Diekwisch T., Luan X., Yang J. (2019). Triple PLGA/PCL scaffold modification including silver impregnation, collagen coating, and electrospinning significantly improve biocompatibility, antimicrobial, and osteogenic properties for orofacial tissue regeneration. ACS Appl. Mater. Interfaces.

[15] Liu J., Chen G., Xu H., Hu K., Sun J.F., Liu M., Zhang F., Gu N. (2018). Pre-vascularization in fibrin Gel/PLGA microsphere scaffolds designed for bone regeneration. NPG Asia Mater..

[16] Niemczyk B., Gradys A., Kolbuk D., Krzton-Maziopa A., Sajkiewicz P. (2019). Crosslinking kinetics of methylcellulose aqueous solution and its potential as a scaffold for tissue engineering. Polymers.

[17] Singh Y.P., Moses J.C., Bhardwaj N., Mandal B.B. (2018). Injectable hydrogels: A new paradigm for osteochondral tissue engineering. J. Mater. Chem. B.

[18] Liu X., Tao J., Liu J., Xu X., Zhang J., Huang Y., Chen Y., Zhang J., Deng D.Y.B., Gou M. (2019). 3D printing enabled customization of functional microgels. ACS Appl. Mater. Interfaces.

[19] Santos-Rosales V., Iglesias-Mejuto A., García-González C.A. (2020). Solvent-free approaches for the processing of scaffolds in regenerative medicine. Polymers.

[20] Choi W.J., Hwang K.S., Kwon H.J., Lee C., Kim C.H., Kim T.H., Heo S.W., Kim J.H., Lee J.Y. (2020). Rapid development of dual porous poly(lactic acid) foam using fused deposition modeling (FDM) 3D printing for medical scaffold application. Mater. Sci. Eng. C.

[21] Sooriyaarachchi D., Minière H.J., Maharubin S., Tan G. (2018). Hybrid additive microfabrication scaffold incorporated with highly aligned nanofibers for musculoskeletal Tissues. Tissue Eng. Regen. Med..

[22] Hiller T., Berg J., Elomaa L., Röhrs V., Ullah I., Schaar K., Dietrich A.C., Al-Zeer M.A., Kurtz A., Hocke A.C. (2018). Generation of a 3D liver model comprising human extracellular matrix in an alginate/gelatin-based bioink by extrusion bioprinting for infection and transduction studies. Int. J. Mol. Sci..

[23] Kwon D.Y., Kwon J.S., Park S.H., Park J.H., Jang S.H., Yin X.Y., Yun J.H., Kim J.H., Min B.H., Lee J.H. (2015). A computer-designed scaffold for bone regeneration within cranial defect using human dental pulp stem cells. Sci. Rep..

[24] Kwon D.Y., Park J.H., Jang S.H., Park J.Y., Jang J.W., Min B.-H., Kim W.D., Lee H.B., Lee J., Kim M. (2017). Bone regeneration by means of a three-dimensional printed scaffold in a rat cranial defect. J. Tissue Eng. Regen. Med..

[25] Park J.H., Kang H.J., Kwon D.Y., Lee B.K., Lee B., Jang J.W., Chun H.J., Kim J.H., Kim M. (2015). Biodegradable poly(lactide-co-glycolide-co-ε-caprolactone) block copolymers—Evaluation as drug carriers for a localized and sustained delivery system. J. Mater. Chem. B.

[26] Hou Q., Grijpma D.W., Feijen J. (2003). Porous polymeric structures for tissue engineering prepared by a coagulation, compression moulding and salt leaching technique. Biomaterials.

[27] Kim M.S., Ahn H.H., Na Shin Y., Cho M.H., Khang G., Lee H.B. (2007). An in vivo study of the host tissue response to subcutaneous implantation of PLGA- and/or porcine small intestinal submucosa-based scaffolds. Biomater..

[28] Yassin M.A., Fuoco T., Mohamed-Ahmed S., Mustafa K., Finne-Wistrand A. (2019). 3D and porous RGDC-functionalized polyester-based scaffolds as a niche to induce osteogenic differentiation of human bone marrow stem cells. Macromol. Biosci..

[29] Witherel C.E., Abebayehu D., Barker T.H., Spiller K.L. (2019). Macrophage and fibroblast interactions in biomaterial-mediated fibrosis. Adv. Health Mater..

