# Supplementary material for: Comparison of Scaffolds Fabricated via 3D Printing and Salt Leaching: In Vivo Imaging, Biodegradation, and Inflammation
Source: Polymers (Basel). 2020 Sep 26;12(10):2210. doi: 10.3390/polym12102210 (PMC7599662; doi:10.3390/polym12102210)
Supplement: Supplementary file 1 [file polymers-12-02210-s001.pdf]

polymers

Supplementary Files

Comparison of Scaffolds Fabricated via 3D Printing and Salt Leaching: In-vivo Imaging, Biodegradation, and Inflammation

Doo Yeon Kwon, Joon Yeong Park, Bun Yeoul Lee, Moon Suk Kim*

[1] Department of Molecular Science and Technology, Ajou University, Suwon, Korea; kdy@ajou.ac.kr (D.Y.K); pjy16@ajou.ac.kr (J.Y.P.); bunyeoul@ajou.ac.kr (B.Y.L.)

* Correspondence; moonskim@ajou.ac.kr (M.S.K.); Tel.: +82-31-219-2608 (M.S.K.)

† Doo Yeon Kwon and Joon Yeong Park are equal first authors.

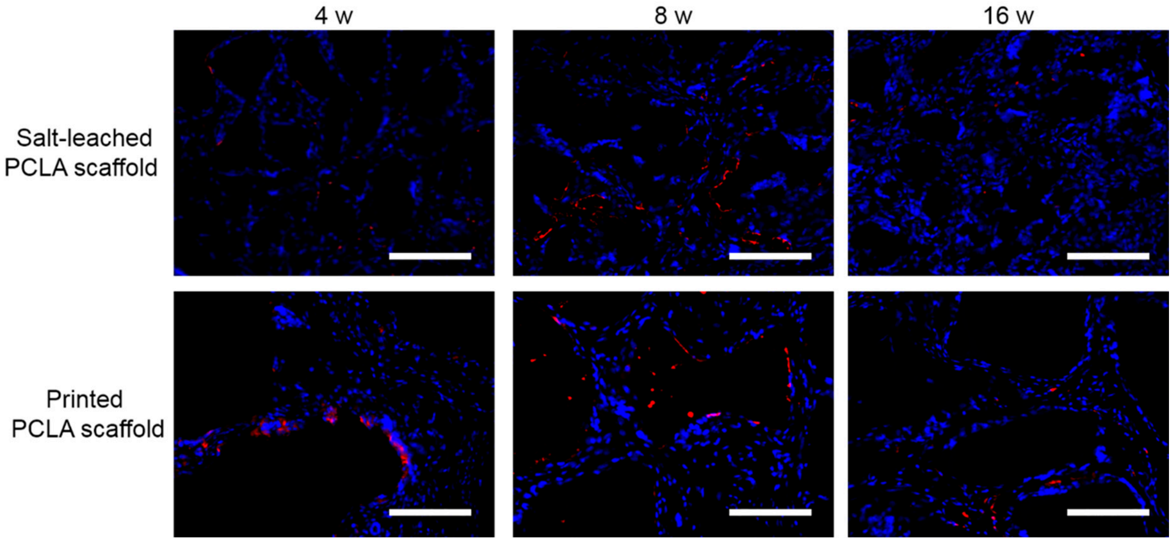

Figure S1. Immunofluorescence ED1 staining images on salt-leached and printed PCLA scaffolds four, eight, and sixteen weeks after implantation. Scale bars = 100 μm.
